# The Selective Localization of Organic Montmorillonite at the Interface and Its Effects on the Micro-Morphology and Properties of Bio-Based Polylactic Acid/Eucommia Ulmoides Gum (PLA/EUG) Blends

**DOI:** 10.3390/polym17070911

**Published:** 2025-03-28

**Authors:** Yipeng Zhang, Kai Wang, Jianing Shen, Luyao Li, Nai Xu, Lisha Pan, Sujuan Pang, Jianhe Liao

**Affiliations:** 1 School of Materials Science and Engineering, Hainan University, Haikou 570228, China; zhangyipeng2013@163.com (Y.Z.); wang_kai126@163.com (K.W.); shenjianing0823@163.com (J.S.); luyaoli1120@yeah.net (L.L.); 2 Hunan Key Laboratory of Near-Space Meteo-Ballon Materials and Technology, Zhuzhou 412003, China; 3 School of Chemistry and Chemical Engineering, Hainan University, Haikou 570228, China; happylisap@hainanu.edu.cn; 4 Hainan Provincial Fine Chemical Engineering Research Center, Hainan University, Haikou 570228, China; psjuan@hainanu.edu.cn

**Keywords:** polylactic acid, Eucommia ulmoides gum, selective localization, organic montmorillonite, micro-morphology, mechanical properties

## Abstract

Highly toughened bio-based polylactic acid (PLA)/*Eucommia ulmoides* gum (EUG) blends were prepared using organic montmorillonite (OMMT) as a compatibilizer through melt-blending. Both the theoretically predicted values and the experimental results confirm that the majority of the OMMT’s nanolayers are selectively localized at the PLA/EUG interface. This localization leads to improved interfacial properties and a more refined morphology of the dispersed EUG phase. By increasing the OMMT content from 0 phr to 2 phr, the notched Izod impact strength of the PLA/EUG/OMMT (85/15/2) blend increases to a maximum value of 44.6 kJ/m^2^. This is significantly higher than the values observed for neat PLA at 3.8 kJ/m^2^ and the PLA/EUG (85/15) blend at 4.7 kJ/m^2^. Moreover, compared to neat PLA and the PLA/EUG (85/15) blend, which exhibit poor tensile ductility, as indicated by their low elongation at break, the PLA/EUG/OMMT blend demonstrates a substantial improvement in its tensile ductility when an appropriate amount of OMMT is added. It is believed that the enhanced toughness of the PLA/EUG/OMMT blends can primarily be attributed to the refinement and more uniform dispersion of the EUG domains, which is caused by the incorporation of OMMT. In addition, the crystalline properties, thermal degradation behavior, and extrudate swell behavior of the PLA/EUG blends with and without OMMT were also evaluated in detail. Finally, the experimental results prove that the PLA/EUG (85/15) blend containing 2 phr of OMMT exhibits the highest impact toughness and tensile ductility, accompanied by improved thermal stability and extrusion stability.

## 1. Introduction

The exhaustion of petroleum resources, along with environmental concerns and problems with resources arising due to the use of traditional petroleum-based polymers, has become more serious. Hence, their replacement with sustainable polymers derived from renewable resources can provide a feasible solution to reduce the dependence on materials from fossil resources. This would also minimize the pollution caused by the heavy use of plastics from petroleum-based sources [1].

Polylactic acid (PLA) is a sustainable polymer, obtained through the direct polycondensation of lactic acid or ring-opening of lactide, wherein both the raw materials are derived from renewable plant resources (e.g., potatoes, corn, etc.) [1]. PLA has some excellent properties, such as high mechanical strength, easy formability, good biodegradability and biocompatibility, etc. Hence, PLA is considered to be one of the most important sustainable bio-based plastics. It has a great potential for applications in many areas, such as medicine, hygiene, agriculture, packaging, single-use items, and mostly to replace petroleum-based plastics [2,3]. However, PLA also has some shortcomings which limit its use in some specific areas. Specially, its inherent brittleness, as evidenced by its poor toughness and ductility, greatly limit the scope of its application [4].

Physical blending of PLA with elastomers and flexible polymers is an efficient and cost-effective methodology for improving its impact toughness or tensile ductility [5]. According to the literature, many kinds of elastomers and flexible polymers can be used as toughening agents to enhance the mechanical performance of PLA. These include natural rubber (NR) [6], ethylene–vinyl acetate (EVA) [7], thermoplastic polyurethane (TPU) [8], polycaprolactone (PCL) [9], poly(butylene succinate) (PBS) [10], poly(butylene adipate-*co*-terephthalate) (PBAT) [11], poly(butylene succinate-*co*-adipate) (PBSA) [12], etc. Among these, natural rubber (NR, *cis*-1,4-polyisoprene), a renewable elastomer derived from *Hevea brasiliensis* [13], is a suitable candidate for use as a toughening modifier for PLA based on the concept of bio-based materials. However, the poor compatibility between PLA and NR fails to achieve the desired toughness when they are simply blended together [6]. Therefore, in recent years, both in situ techniques, namely chemical compatibilization (e.g., dynamic vulcanization [14]), and the utilization of modified NR (e.g., glycidyl methacrylate-grafted NR [15], PLA-NR-PLA [16], and PMMA-grafted NR [17] etc.) as a toughener or compatibilizer have been used in the PLA/NR blending system to improve their interfacial compatibility, thus achieving a better mechanical performance with PLA/NR blends.

Other than natural rubber, *Eucommia ulmoides* gum (EUG) is also an important type of bio-based elastomer, obtained from the seeds, bark, roots, and leaves of *Gutta percha*. The main component of EUG is *trans*-1,4-polyisoprene, an isomer of natural rubber [18]. Compared to NR, the semi-crystalline nature of EUG means it combines the high elasticity of rubbers and the good plasticity of plastics, which enables its use in many fields. Therefore, as a renewable bio-based material, EUG is a potential candidate for toughening PLA.

Presently, a few research works on the toughening of PLA with bio-based EUG have been reported. For example, Kang et al. [19] prepared binary PLA/EUG blends with varying EUG contents through a melt-blending process. DMA, DSC, and SEM analyses indicated that PLA and EUG were thermodynamically immiscible. The PLA/EUG (90/10) and (85/15) blends exhibited a maximum notched Izod impact strength of 21.1 kJ/m^2^ and a maximum elongation at break of 81%, compared to 2.4 kJ/m^2^ and 5.0%, respectively, for neat PLA.

In order to improve the interfacial compatibility between the PLA matrix and the EUG component, Wang et al. [20] first modified the polarity of EUG resin through epoxidation. Further, a series of PLA/epoxidized EUG (EEUG) thermoplastic vulcanizates (TPVs) were prepared using a dynamic vulcanization technique. The authors proposed that both EEUG and PLA could form macromolecular radicals through radical-seizing hydrogen atoms in the presence of a DCP initiator. The two macromolecular radicals interacted with each other to form graft copolymers (EEUG-*g*-PLA). EEUG-*g*-PLA acted as an interfacial transition layer in the TPVs and enhanced the compatibility between the two phases. The PLA/EEUG TPV with 40 wt% EEUG was super tough, with a notched Izod impact strength of 47.3 kJ/m^2^. Later, Wang et al. [21] introduced polar groups into EUG through bulk-free radical polymerization with glycidyl methacrylate (GMA) and improved its compatibility with PLA. Then, the modified EUG was blended with PLA to obtain PLA/modified EUG TPVs with improved interfacial properties, super toughness, and shape memory properties through an in situ dynamic vulcanization technique. It was confirmed that the PLA/modified EUG TPVs exhibited a co-continuous phase structure and improved interfacial properties. With an increase in the proportion of modified EUG to 40 wt%, the notched Izod impact strength of the PLA/modified EUG TPV increased to 54.8 kJ/m^2^, 20 times more than that of neat PLA (2.4 kJ/m^2^).

Compared to in situ reactive compatibilization, the use of physical compatibilizers has been another facile and efficient tactic for improving the interfacial compatibility and mechanical performance of incompatible polymer blending systems without bringing a significant change in the macromolecular structures and rheological behaviors. In recent years, the incorporation of nanoparticles as a novel physical compatibilizer (an interfacial modifier) into PLA blends has been reported, which include SiO_2_ [22], TiO_2_ [23], Fe_3_O_4_ [24], CNTs [25], graphene [26], etc. Due to their nano-size and high adsorption capacity, these nanoparticles can be selectively distributed in the dispersed phase or the continuous phase or at the interfaces of the PLA blends, which affects the phase morphology and interfacial properties of the blends. Hence, the selective location of nanofillers in a blending system may become an effective strategy for tailoring the micro-structures and mechanical performance of PLA blends.

Montmorillonite (MMT, a kind of clay) [27,28], owing to its layered silicate structure and high aspect ratio, has been used as an active interfacial modifier for immiscible PLA blends and is gaining more and more attention. For example, Reza Salehiyan et al. [29] investigated the distribution of MMT nanoparticles at the interface of a polylactide/poly(butylene succinate) (PLA/PBS) blend, prepared using multifunctional epoxide as a reactive compatibilizer, and studied their impact on the development of the microstructure. Two types of organic MMT (OMMT), one more hydrophilic (Cloisite^®^ 30B (C30B)) and another one more hydrophobic (Betsopa^TM^ (BET)), were used at different OMMT concentrations. It was confirmed that both C30B and BET were preferentially located mostly at the interface and in the PLA matrix. However, random agglomerations were found within the blends when BET was used, which can be explained by its lower stabilization efficiency. This could be attributed to the less favorable enthalpic interactions between the organic surfactant in BET and the blend matrix as compared to that between the organic surfactant in C30B and the blend matrix. Zhu et al. [30] prepared PCL/PLA/MMT nanocomposites, in which the influences of MMT on the microphase dispersions and interfacial interactions were studied and compared with those of the blank PCL/PLA blend. When MMT particles were introduced, these MMT nanolayers preferentially localized at the PCL/PLA interface. The results showed that the introduction of MMT not only increased the interfacial interactions in the PCL/PLA blend significantly but also caused a significant reduction in the particle size of the dispersed phase and prevented coalescence between the phases during melt mixing. Yu et al. [31] prepared PLA/PGA nanocomposites by introducing MMT modified with octadecyl trimethyl ammonium chloride (OTAC). OTAC was successfully intercalated into the MMT interlayers through cation exchange, thus increasing the interlayer spacing and decreasing the surface free energy. More importantly, the interfacial compatibility increased significantly after the introduction of -NH_2_ groups into the MMT and the formation of hydrogen bonds with the -OH groups of PLA and PGA. With an increase in the OMMT content to 3%, the tensile strength and modulus of the PLLA/PGA nanocomposites increased by 110% and 70%, respectively.

To the best of our knowledge, there are only a few reports on the compatibilization of nanoparticles for PLA/EUG blends. In this study, a facile melt-blending technique was employed to develop PLA/EUG blends with enhanced toughness, in which OMMT was used as an efficient physical compatibilizer. The influences of the OMMT on the micro-morphology, mechanical performance, crystallization, and thermal properties of the PLA/EUG blends were researched in detail. The mechanisms of compatibilization and toughness in the PLA/EUG blends resulting from the incorporation of OMMT were analyzed and discussed. Finally, the impact of OMMT on the extrudate swell behavior of the PLA/EUG melt was also evaluated through a capillary extrusion experiment. The PLA/EUG blends obtained, with a more balanced performance, especially enhanced toughness, will have promising application prospects in the field of eco-friendly and renewable general plastic products, such as shopping bags, packaging films, tableware, plastic parts for toys, electrical appliance shells, etc.

## 2. Materials and Methods

### 2.1. Raw Materials

PLA resin (4032D), with a density of 1.25 g/cm^3^ and an MFR of 3.3 g/10 min (190 °C, 2.16 kg loading), was purchased from Nature Works LLC (Minnetonka, MN, USA). The *Eucommia ulmoides* gum (EUG, *trans*-1,4-polyisoprene), with a density of 0.91 g/cm^3^, was supplied by Western Hunan Laodie Biotechnology Co., Ltd. (Zhangjiajie, China).

Organic montmorillonite (OMMT, 1.44PSS) modified with dihydrogenated tallow dimethyl ammonium/siloxane was obtained from Nanocor Co. (Arlington Heights, IL, USA).

**Figure 1:**
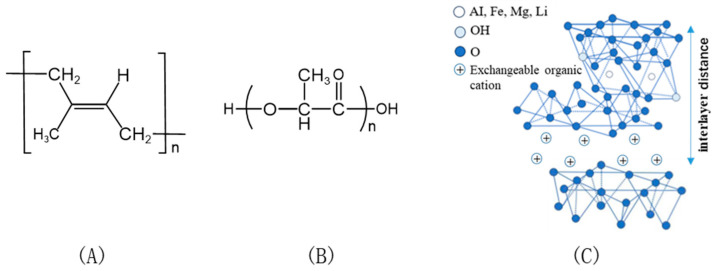
Chemical structures of (**A**) EUG, (**B**) PLA, and (**C**) OMMT.

Figure 1 shows the chemical structures of EUG, PLA, and OMMT.

### 2.2. Sample Preparation

The raw materials PLA and EUG were dried in a vacuum oven at 50 °C for 8 h. OMMT was dried in an air oven at 100 °C for more than 5 h. Then, the PLA/EUG/OMMT (85/15/x) blends were prepared using a co-rotating twin screw extruder (SHJ-20, Nanjing Giant Co., Ltd., Nanjing, China), in which the diameter and the length–diameter (L/D) ratio of the extruder screw were 20 mm and 40:1, respectively. During melt extrusion, the screw speed was maintained at 100 rpm, and the temperatures from the hopper to the die were sequentially set at 150 °C, 190 °C, 190 °C, 190 °C, 190 °C, and 180 °C. The mass ratios of the PLA/EUG/OMMT (85/15/x) blends were 85/15/0, 85/15/1, 85/15/2, 85/15/4, and 85/15/6, where x denotes the amount of OMMT in parts per hundred of total polymer resins (phr). The PLA/EUG/OMMT (85/15/x) mass ratios are listed in Table 1.

**Figure 2:**
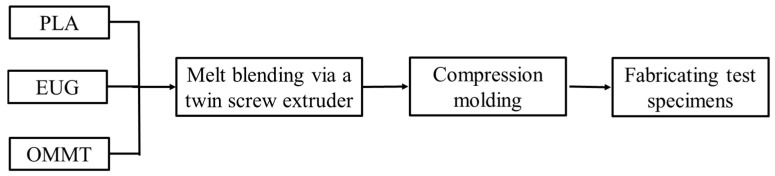
Flowchart of blending processing and preparation of test specimens.

The obtained extrudates were compression-molded through hot pressing at 210 °C and 15 MPa for 8 min. Then, the closed molds containing the in-mold melt were quickly transferred into a water-cooling system at a clamping pressure of 15 MPa for cooling and solidification. Thus, compression-molded sheets with a thickness of ca. 1 mm or 4 mm were obtained. Dumbbell-shaped specimens for tensile testing were prepared using die cutting from the sheets of a 1 mm thickness. For the dumbbell specimens, the width and length of the narrow section were 4 and 30 mm, respectively. The notched impacted specimens for the Izod notched impact strength test were prepared from the sheets of a 4 mm thickness using a universal specimen-making machine. The dimensions of the notched impact specimens were 80 mm × 10 mm × 4 mm, and the notches were of type A. Figure 2 presents a flowchart of the blending processing and preparation of the test specimens.

### 2.3. Contact Angle Measurements

A contact angle measuring instrument (DropMeter Experience A-300, Haishu Maishi Inspection Technology Co., Ltd., Ningbo, China) was used to test the contact angle *θ* values of the neat PLA and EUG samples with specific solvents. The samples (50 mm × 50 mm × 2 mm) were prepared through compression molding. The molding temperature and pressure were 210 °C and 15 MPa, respectively. The liquids chosen for calibration were deionized water (H_2_O) and diiodomethane (CH_2_I_2_). The experiments were conducted at an ambient temperature of 25 °C. Each test result was the average of no less than five valid values for each sample.

### 2.4. Transmission Electron Microscopy (TEM)

The dispersion of OMMT in the PLA/EUG/OMMT blends was investigated through TEM (JEM-2100, JOEL, Akishima, Japan) at an accelerating voltage of 200 kV. Ultrathin sections of the samples with a thickness of ca. 80 nm were prepared using a freezing microtome.

### 2.5. Scanning Electron Microscopy (SEM)

The cryo-fractured surfaces and notched impact-fractured surfaces of the specimens were examined through SEM (Verios G4 UC, Thermo Fisher Scientific Inc., Waltham, MA, USA) at an accelerating voltage of 5 kV. The samples for testing were cryo-fractured after immersion in liquid nitrogen for 5 min. All of the fractured surfaces were sputter-coated with a thin layer of gold to prevent electrical discharge during the SEM observation.

### 2.6. Mechanical Performance

Tensile testing was performed using electronic tensile testing equipment (WDW-1, Yinuo Century Test Instrument Co., Ltd., Jinan, China) in accordance with ASTM D638-22 [32], wherein the tensile yield strength, tensile modulus, and elongation at break were measured at a crosshead velocity of 5 mm/min. The mean value was calculated from at least five valid values for each data point and reported along with its standard deviation.

The notched Izod impact strength was measured using a pendulum impact tester (XJU-5.5, Dahua Machine Co., Ltd., Chengde, China) in accordance with ASTM D256-24 [33], wherein the mean value was calculated from at least five valid values for each data point, along with the standard deviation.

### 2.7. X-Ray Diffraction (XRD) Analysis

X-ray diffractometry (D8 ADVANCE, Bruker, Karlsruhe, Germany) was used to characterize the crystal structures of the PLA/EUG/OMMT samples. The XRD data were recorded in the range of 5° ≤ 2θ ≤ 35°at a scanning speed of 2°/min (CuKα radiation, λ = 1.54 Å). In addition, the intercalated structure of the OMMT nanolayers dispersed in the samples was also characterized using XRD in the range of 1° ≤ 2θ ≤ 10° at a scanning speed of 1° /min.

### 2.8. Differential Scanning Calorimetry (DSC)

Differential scanning calorimetry (DSC) was carried out using a DSC instrument (Q100, TA Instruments, New Castle, DE, USA), and the DSC thermograms were used to determine the crystallinities of the samples. The analysis was conducted according to ASTM E793-06(2018) by heating them from 20 °C to 200 °C at a heating rating of 10 °C/min in a nitrogen atmosphere [34].

### 2.9. Thermogravimetric Analysis (TGA) Measurement

A TGA thermal analyzer (Q600, TA Instruments, New Castle, DE, USA) was used to study the influence of the OMMT content on the thermal degradation behaviors of the PLA/EUG blends from 30 °C to 700 °C at a heating rating of 20 °C/min in a nitrogen atmosphere with a nitrogen gas flow rate of 50 mL/min.

### 2.10. Extrudate Swell Ratio

A single screw extruder (LSJ20, Ke-chuang Rubber Plastics Machinery Ltd., Shanghai, China), equipped with a capillary die, was used to evaluate the extrudate swell behaviors of the PLA/EUG/OMMT blends with different OMMT contents. The diameter and L/D ratio of the screw were 20 mm and 25:1, and the internal diameter and the L/D ratio of the capillary die were 1.27 mm and 20:1, respectively. The setting temperatures of the single screw extruder from the hopper to the die were 125 °C, 190 °C, 190 °C, and 190 °C, sequentially. The screw speeds were maintained at 10 rpm and 20 rpm.

The extrusion swell behavior of a polymer melt, also known as the die swell behavior, can be simply evaluated as the extrudate swell ratio (*ESR*), as shown in Equation (1).

(1) $$ESR=\frac{D_{sample}}{D_{capillary}}$$

where *D*_sample_ and *D*_capillary_ represent the diameter of the extrudate (mm) and the internal diameter of the capillary die (1.27 mm).

## 3. Results and Discussion

### 3.1. The Localization of OMMT in the PLA/EUG Blend

The wetting coefficient $\omega_{\alpha }$ (according to Young’s equation) is often used to predict the location of inorganic fillers in immiscible polymer blends [35,36]:

(2) $$\omega_{\alpha }=\frac{\gamma_{\left(PLA-OMMT\right)}-\gamma_{\left(EUG-OMMT\right)}}{\gamma_{\left(PLA-EUG\right)}}$$

where *γ*_(PLA-OMMT)_ represents the interfacial energy between PLA and OMMT, *γ*_(EUG-OMMT)_ represents the interfacial energy between EUG and OMMT, and *γ*_(PLA-EUG)_ represents the interfacial energy between PLA and EUG. When the calculated $\omega_{\alpha }$ < −1, the OMMT nanoparticles tend to be distributed in the PLA matrix; when $\omega_{\alpha }$ > 1, the OMMT nanoparticles tend to be distributed in the EUG domains; and when −1 < $\omega_{\alpha }$ < 1, the OMMT nanoparticles tend to be distributed at the interface of the PLA matrix and the EUG domains.

The interfacial energies between component pairs can be calculated by applying the harmonic-mean equation and the geometric-mean equation [36,37].

The harmonic-mean equation is as follows:

(3) $$\gamma_{ij}=\gamma_{i}+\gamma_{j}-4\left(\frac{\gamma_{i}^{d}\gamma_{j}^{d}}{\gamma_{i}^{d}+\gamma_{j}^{d}}+\frac{\gamma_{i}^{p}\gamma_{j}^{p}}{\gamma_{i}^{p}+\gamma_{j}^{p}}\right)$$

The geometric-mean equation is as follows:

(4) $$\gamma_{ij}=\gamma_{i}+\gamma_{j}-2\left(\sqrt{\gamma_{i}^{d}\gamma_{j}^{d}}+\sqrt{\gamma_{i}^{p}\gamma_{j}^{p}}\right)$$

where the subscripts *i* and *j* represent the PLA and EUG components, respectively, and γ is the surface energy. The superscripts d and p denote the dispersive (nonpolar) and polar parts of the surface energy, respectively.

Since it is difficult to directly obtain the surface energies experimentally, contact angle measurements are usually made to calculate the surface energies of the polymers. The relation between the contact angle and the surface energy is given by the Owens–Wendt equation [38]:

(5) $$\gamma_{L}\left(1+\cos\theta \right)=2\sqrt{{\gamma_{i}^{d}\gamma }_{L}^{d}}+2\sqrt{\gamma_{i}^{p}\gamma_{L}^{p}}$$

where $\gamma_{L}$ represents the surface energy of the calibration liquid, and *θ* represents the measured contact angle between the calibration liquid and the polymer.

The surface energies of solids and liquids can be decomposed essentially into the sum of the different forms of molecular action on the surface, that is, the sum of the dispersive energy part and the polar energy part [37].

(6) $$\gamma_{i}=\gamma_{i}^{d}+\gamma_{i}^{p}$$

Therefore, the surface energy $\gamma_{i}$ of the polymer (EUG/or PLA) could be calculated from $\gamma_{i}^{d}$ and $\gamma_{i}^{p}$. In the same way, the surface energy $\gamma_{L}$ of the calibration liquid could also be calculated from $\gamma_{L}^{d}$ and $\gamma_{L}^{p}$.

In this study, deionized water (H_2_O) and diiodomethane (CH_2_I_2_) were used as the calibration liquids. Table 2 lists the surface energy parameters of the calibration liquids and OMMT from previous reports in the literature. Meanwhile, the contact angles of deionized H_2_O and CH_2_I_2_ on the surfaces of the PLA and EUG samples, respectively, measured through contact angle experiments, are also listed in Table 2. By applying the above-mentioned parameters, the surface energies of the PLA and EUG components at room temperature (25 °C) were calculated using Equations (5) and (6) and extrapolated to the processing temperature (190 °C) by using the temperature coefficients (−d*γ*/d*T*) reported in the literature [39], as shown in Table 2.

Based on the harmonic-mean equation and the geometric-mean equation, the interfacial energies between the components and the corresponding wetting coefficients were calculated at the processing temperature (190 °C) and are listed in Table 3. Since the values of the wetting coefficients calculated from both the harmonic-mean equation and the geometric-mean equation are between −1 and 1, it can be said that the OMMT nanolayers are located at the PLA/EUG interfaces [36].

**Figure 3:**
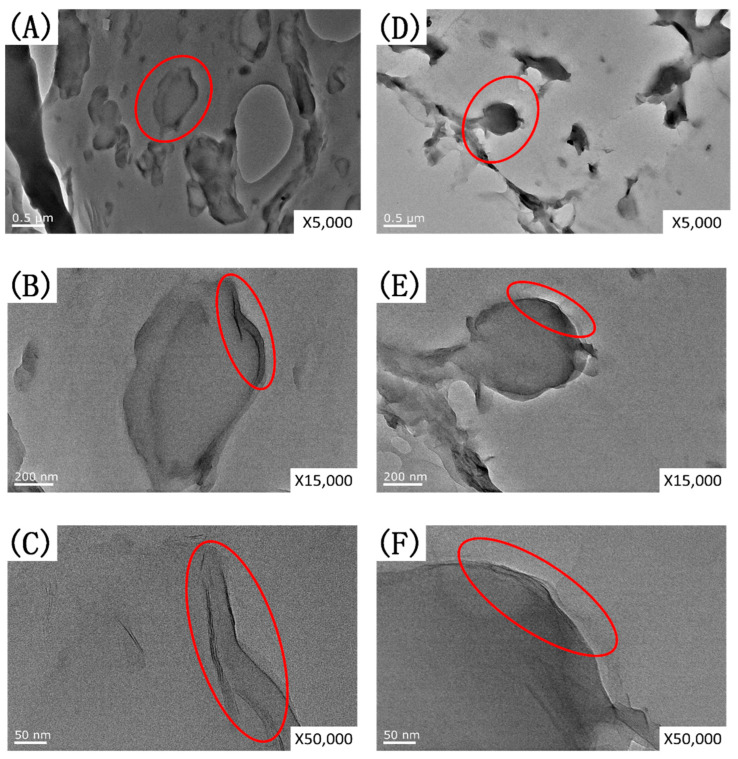
TEM micrographs for (**A**–**C**) PLA/EUG/OMMT (85/15/2) and (**D**–**F**) PLA/EUG/OMMT (85/15/4) samples with various magnifications.

A TEM analysis was further used to investigate the selective location of OMMT in the PLA/EUG blends. TEM images of the PLA/EUG/OMMT (85/15/2) and (85/15/4) samples at different magnifications (×5 k, ×15 k, and ×50 k) are shown in Figure 3.

From Figure 3, it is evident that in the PLA/EUG/OMMT (85/15/x) blends, the distribution of the dispersed EUG phase in the PLA matrix exhibits a granular morphology. In particular, a large number of OMMT nanolayers are distributed at the interface between EUG and PLA, which is consistent with our theoretical calculations. Theoretically, in the PLA/EUG blends, the selectively distributed OMMT nanolayers at the PLA/EUG interface could reduce the interfacial tension, leading to a decrease in the size of the dispersed EUG droplets. Meanwhile, the OMMT at the interface functions not only as an emulsifier but also effectively encapsulates the EUG dispersion droplets, thereby preventing their re-aggregation.

The high-magnification TEM images (see Figure 3C,F) reveal an intercalated structure of OMMT nanolayers in the PLA/EUG/OMMT blends. An XRD analysis further characterized the intercalated structure of the OMMT nanolayers dispersed in the PLA/EUG/OMMT blends. The ordered array of montmorillonite silicate layers shows diffraction peaks at their corresponding positions, with which the interlayer spacing of montmorillonite can be calculated using Bragg’s equation (Equation (7)).

2 × *d* × sin*θ* = *n* × *λ* (7)

where *λ* refers to the wavelength of X-ray diffraction (*λ* = 1.54 Å), *d* stands for the interlayer spacing between the OMMT layers (the spacing between diffracting lattice planes), and *θ* is the corresponding diffraction peak value.

**Figure 4:**
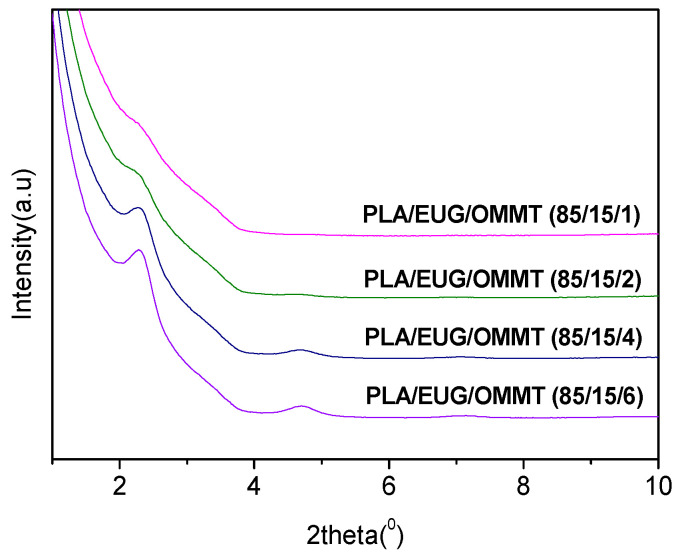
XRD patterns of PLA/EUG/OMMT (85/15/x) samples.

Figure 4 shows the X-ray diffraction patterns of the PLA/EUG/OMMT blends. The (*d*_001_) diffraction peak of OMMT in the PLA/EUG/OMMT blends appears at 2*θ* = 2.28°, which corresponds to an interlayer spacing of 3.87 nm in the OMMT. However, OMMT powder shows a (*d*_001_) diffraction peak at 2*θ* = 3.56° which corresponds to an interlayer spacing of 2.48 nm. This indicates that a small number of polymer chains have interpolated into the OMMT layers, with the formation of an intercalated structure in the PLA/EUG/OMMT blend.

### 3.2. Micro-Morphology and Mechanical Properties

**Figure 5:**
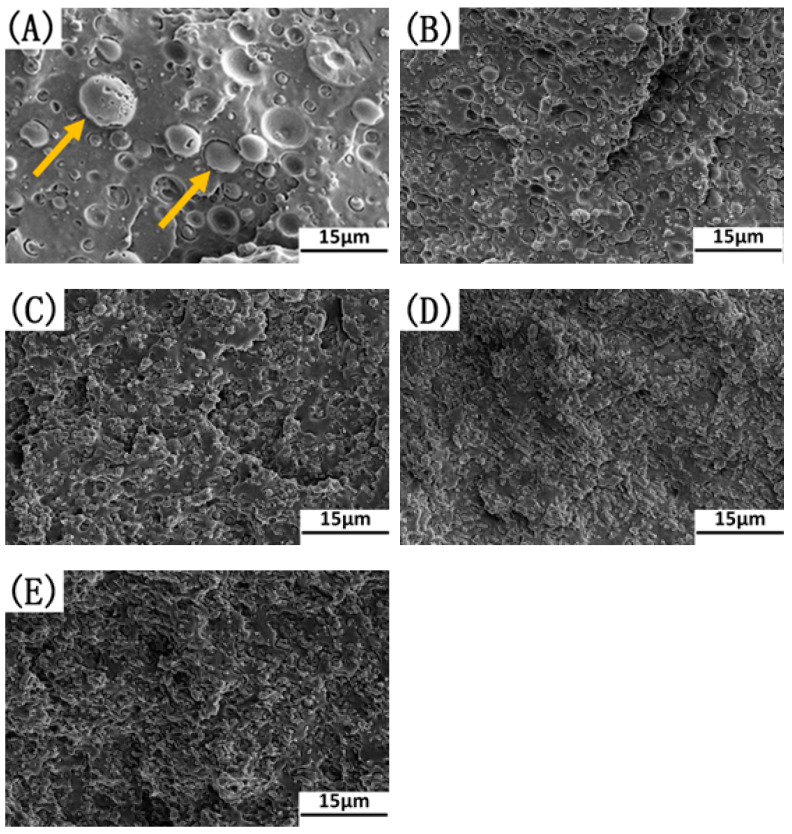
SEM micrographs of cryo-fractured surfaces of PLA/EUG/OMMT samples with various OMMT contents: (**A**) PLA/EUG (85/15), (**B**) PLA/EUG/OMMT (85/15/1), (**C**) PLA/EUG/OMMT (85/15/2), (**D**) PLA/EUG/OMMT (85/15/4), and (**E**) PLA/EUG/OMMT (85/15/6).

The influence of the OMMT content on the micro-morphology of the PLA/EUG blends was investigated using SEM. The SEM images of the PLA/EUG/OMMT (85/15/x) samples are presented in Figure 5. In the case of the binary blend of PLA/EUG, a typical immiscible two-phase morphology with poor interfacial adhesion and large EUG domains is observed in Figure 5A. However, with an increase in the OMMT content, the domain size of the dispersed EUG particles decreases considerably, and the PLA/EUG interface becomes more and more blurred (see Figure 5B–E).

**Figure 6:**
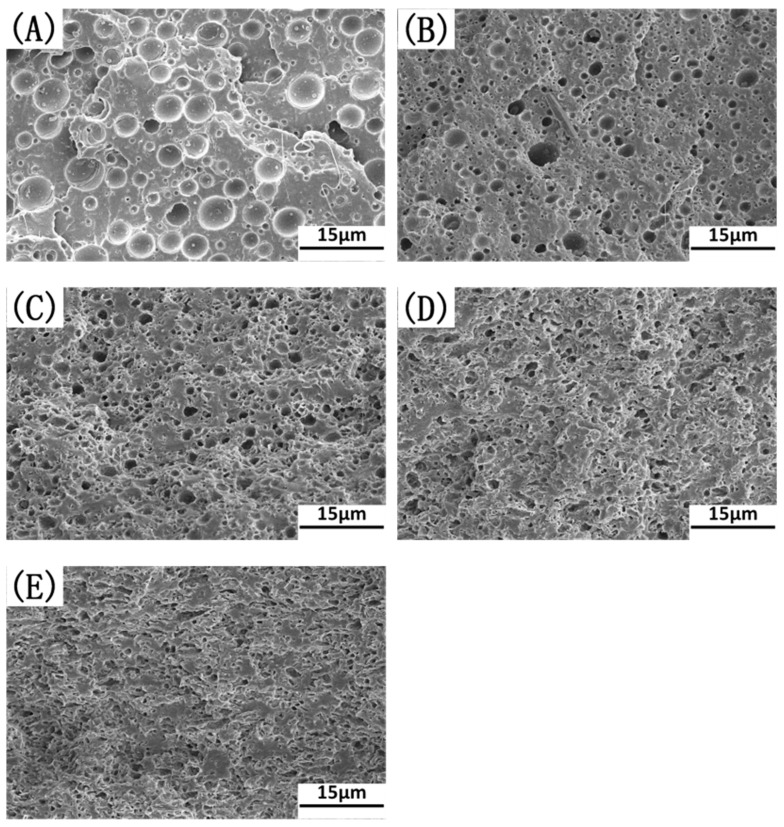
SEM micrographs of the cyclohexane-etched cryo-fractured surfaces of PLA/EUG/OMMT samples with various OMMT contents: (**A**) PLA/EUG (85/15), (**B**) PLA/EUG/OMMT (85/15/1), (**C**) PLA/EUG/OMMT (85/15/2), (**D**) PLA/EUG/OMMT (85/15/4), and (**E**) PLA/EUG/OMMT (85/15/6).

**Figure 7:**
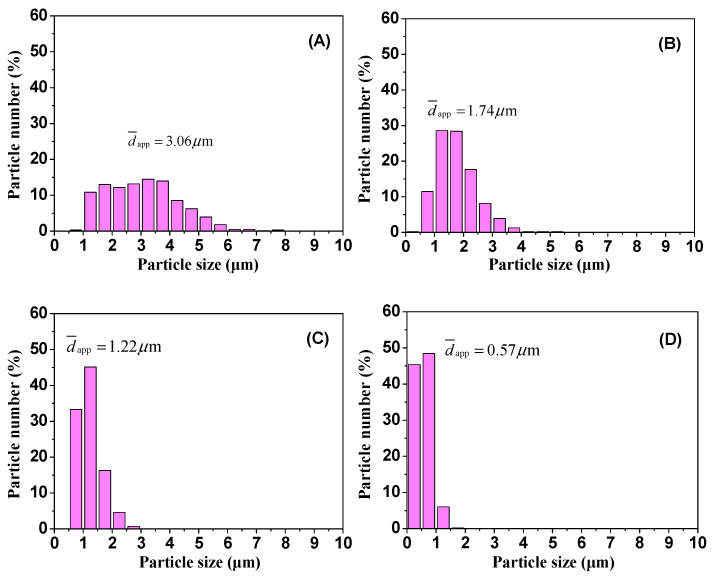
Distribution of EUG particle size of PLA/EUG/OMMT samples with various OMMT contents: (**A**) PLA/EUG (85/15), (**B**) PLA/EUG/OMMT (85/15/1), (**C**) PLA/EUG/OMMT (85/15/2), and (**D**) PLA/EUG/OMMT (85/15/4).

The SEM images of the cyclohexane-etched cryogenically fractured surfaces of the PLA/EUG/OMMT samples with various OMMT contents in Figure 6 present a better view of the morphology of the EUG domains. At least 500 randomly selected EUG particles for each blend sample were measured to obtain the average size of the EUG particles using particle size measurements and statistics software (Nano Measurer 1.2, Fudan University, Shanghai, China). The diameter distribution and the average apparent particle size (${\bar{d}}_{app}$) of the EUG particles are presented in Figure 7. It is evident that with an increase in the OMMT content, ${\bar{d}}_{app}$ decreases by several times, accompanied by narrowing of the particle size distribution. For example, with an increase in the OMMT content from 0 phr to 2 phr, the value of ${\bar{d}}_{app}$ decreases from 3.06 μm to 1.22 μm. With a further increase in the OMMT content to 4 phr, the value of ${\bar{d}}_{app}$ drops sharply to 0.57 μm.

It is well known that in an ideal blend model, the dispersed phase particles are spherical, and the particle size distribution is monodisperse. While its blending component ratio is constant, with the particle size in the dispersed phase reducing from *r*_0_ μm to *r*_1_ μm, the number of particles in the dispersed phase (*n*_1_) increases to a multiple of (*r*_0_/*r*_1_)^3^ of the number of particles in the dispersed phase (*n*_0_) in the initial blend. Obviously, as for the PLA/EUG/OMMT (85/15/x) blending system, a decrease in the EUG particle size can cause a significant increase in the number of EUG particles. This will be accompanied by a significant increase in the specific surface area of the dispersed EUG particles. In this situation, these dispersed EUG particles with smaller particle sizes will help to induce the plastic deformation of the PLA matrix to a large extent. Meanwhile, the increase in the number of EUG particles, coupled with their larger specific surface areas, caused by the refinement of the phase sizes, may increase the probability of crazing. Therefore, the PLA/EUG blend is expected to have improved ductility or toughness when tailoring the micro-morphology of the blend with the addition of OMMT.

To sum up, during the melt-blending process, the OMMT nanolayers selectively located at the PLA/EUG interface can reduce the interfacial tension greatly, leading to refinement of the EUG domains. At the same time, the higher concentration of OMMT nanolayers at the interface can also effectively prevent the re-agglomeration of the EUG droplets. Evidently, due to the localization of OMMT selectively at the PLA/EUG interface, OMMT can be used as an efficient compatibilizer (an interfacial modifier) for PLA/EUG blends. Thus, a series of PLA/EUG/OMMT blends containing finer dispersed EUG particles with a narrowed particle size distribution were successfully prepared using the melt-blending method.

**Figure 8:**
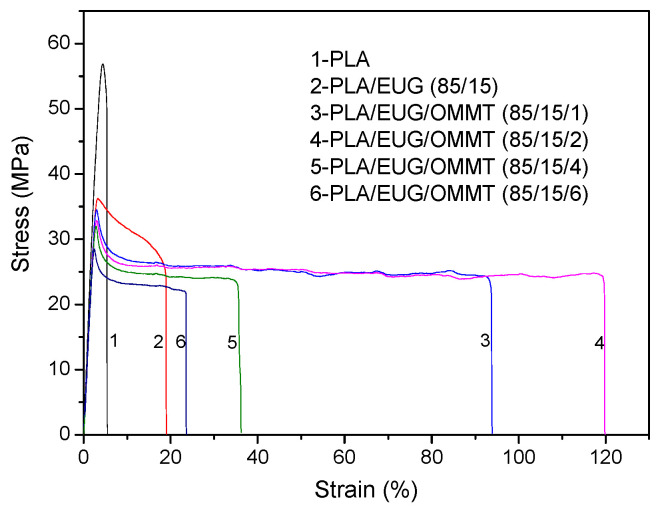
Representative tensile stress–strain curves of PLA and PLA/EUG/OMMT (85/15/x) samples.

The tensile properties of the neat PLA and PLA/EUG/OMMT (85/15/x) samples are listed in Table 4, and the corresponding typical stress–strain curves are shown in Figure 8. It is evident from Table 4 that after adding 15 wt% EUG into PLA, the elongation at break of the binary PLA/EUG sample increases marginally from 5.3% for neat PLA to 18.8%. As expected, after the introduction of 1 phr of OMMT into the PLA/EUG blend, the elongation at break of the PLA/EUG/OMMT (85/15/1) sample drastically increases to 96.6%. With an increase in the OMMT content to 2 phr, the elongation at break further increases to 102.8%. It should be noted that necking and stress-whitening phenomena occurred simultaneously when the PLA/EUG specimens with and without OMMT were cold-drawn under tensile stress. Therefore, the tensile deformation behaviors of the PLA/EUG and PLA/EUG/OMMT samples could be attributed to crazing, along with a shear yielding mechanism. The results show that the binary PLA/EUG blend has low tensile ductility due to its poor interfacial compatibility and larger EUG domain size, with uneven dispersion of the EUG phase. This tends to accelerate the craze-to-microcrack transition and results in fracture at low elongation. In comparison, the refinement of the dispersed EUG particles and a more uniform dispersion of the EUG phases occurred when OMMT was incorporated into the blend. This could largely enhance the crazing termination and shear yielding abilities of the PLA matrix. As a result, the PLA/EUG/OMMT (85/15/1) and (85/15/2) samples displayed a better tensile ductility than that of the binary PLA/EUG (85/15) blend. However, it is evident from Table 4 that the elongation at break exhibits a significant decrease when the OMMT content exceeds 2 phr. This can be attributed to the strong tendency of OMMT nanoparticles to agglomerate when an excessive amount of OMMT particles is blended with the PLA/EUG melt. As a result, these agglomerated OMMT particles became points of stress defects that caused fracturing of the PLA/EUG/OMMT samples at lower elongation.

It has to be pointed out that with an increase in the OMMT content, the tensile yield strength of the PLA/EUG/OMMT (85/15/x) samples constantly decreases. This phenomenon can be attributed to the following reasons: Due to the compatibilization effect of the OMMT, the size of the dispersed EUG particles continuously decreases with an increase in the OMMT content, as shown in Figure 6. When the blend sample is subjected to an external tensile force, the refined EUG particles, owing to their smaller size, can induce a relatively higher stress concentration in the surrounding matrix. This will render the matrix more prone to shear yielding and result in the decreased shear yield strength of the blend sample. Additionally, with the incorporation of rigid OMMT nanolayers, the tensile modulus of the PLA/EUG/OMMT samples does not exhibit an obviously increasing trend. This is because most of the OMMT nanolayers are distributed at the PLA/EUG interface rather than in the PLA matrix (see Figure 3), thus resulting in an insignificant reinforcing effect of the OMMT nanolayers on the PLA matrix in terms of the tensile modulus.

**Figure 9:**
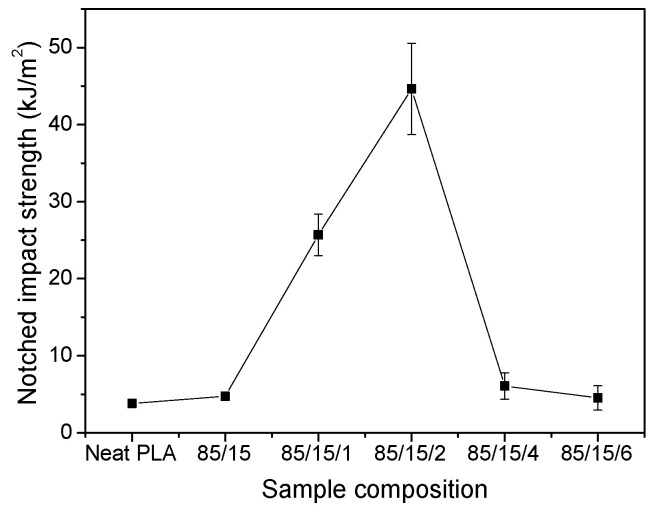
Notched Izod impact strengths of PLA/EUG/OMMT (85/15/x) samples.

Figure 9 shows the notched Izod impact strengths of the neat PLA and PLA/EUG/OMMT (85/15/x) samples. Owing to its brittle nature, the neat PLA sample shows very low impact resistance, wherein its notched strength is as low as 3.8 kJ/m^2^. However, with the introduction of 15 wt.% EUG as a toughness modifier into the PLA matrix, the notched impact strength of the binary PLA/EUG (85/15) sample increases slightly to 4.7 kJ/m^2^. This poor toughening effect of EUG on the PLA can be attributed to the low compatibility between the EUG domains and the PLA matrix. In Figure 9, the notched impact strength shows a sharp increase with the introduction of a moderate amount of OMMT. However, with further increases in the OMMT content, this decreases drastically. This result is consistent with the relationship between tensile ductility and OMMT content. For example, the notched impact strength increases sharply from 4.7 kJ/m^2^ for the binary sample to 25.7 kJ/m^2^ as the OMMT content increases from 0 to 1 phr. Furthermore, on the addition of 2 phr of OMMT, the notched impact strength increases to 44.6 kJ/m^2^, an increase of nearly 849% compared with the value for the binary sample. However, further increases in the OMMT content to 4 phr and 6 phr result in a correspondingly significant decrease in the notched impact strength, to 6.1 kJ/m^2^ and 4.5 kJ/m^2^, respectively.

**Figure 10:**
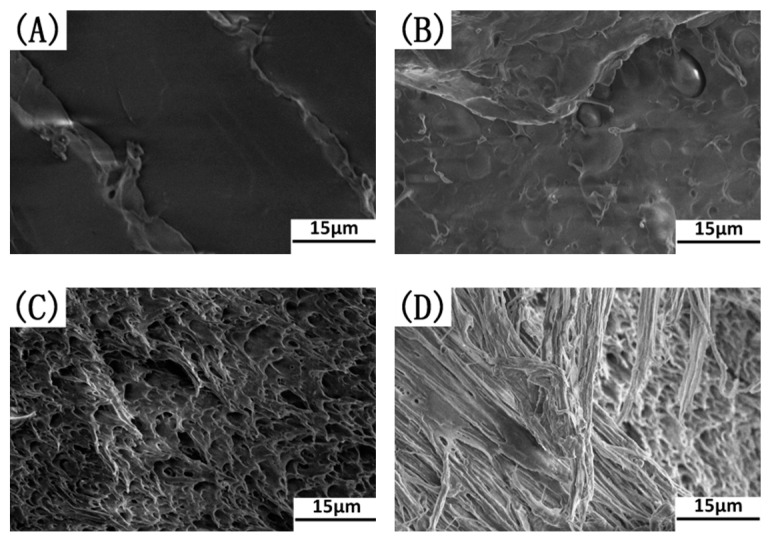

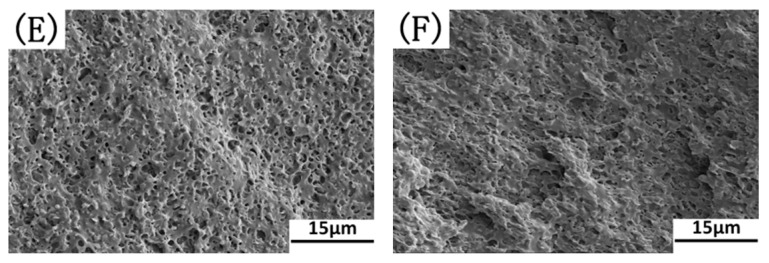
SEM micrographs of notched impact-fractured surfaces of neat PLA and PLA/EUG/OMMT samples: (**A**) neat PLA, (**B**) PLA/EUG (85/15), (**C**) PLA/EUG/OMMT (85/15/1), (**D**) PLA/EUG/OMMT (85/15/2), (**E**) PLA/EUG/OMMT (85/15/4), and (**F**) PLA/EUG/OMMT (85/15/6).

Figure 10 shows SEM images of the notched impact-fractured surfaces of these samples. The SEM image shows that the impact-fractured surface of the neat PLA sample was very smooth and glassy, characteristic of brittle fractured behavior (see Figure 10A). This can be attributed to the brittle nature of PLA materials. Generally, brittle polymers usually show catastrophic fractures since they lack energy-absorbing mechanisms with which they could dissipate energy during the failure process. As shown in Figure 10B, for the binary sample, the fractured surface shows much less plastic deformation, indicating a semi-brittle failure through fast crack propagation. The low impact resistance offered by the binary blend can be attributed to the poor interfacial compatibility in PLA/EUG and the large domain size of EUG, with an uneven phase dispersion. Interestingly, in Figure 10C,D, the fractured surfaces of the PLA matrix show excessive plastic deformation after the addition of 1 phr and 2 phr of OMMT. It is confirmed from Figure 5 that OMMT has a strong interfacial compatibilization effect on the PLA/EUG blends, resulting in the refinement of the dispersed EUG particles and the more uniform phase dispersion of EUG in the matrix. The refined EUG domains can promote plastic deformation of the matrix surrounding the dispersed EUG phases through the mechanism of crazing with shear yielding. Plastic deformation of the polymer matrix was the main energy-absorbing process which dissipated energy during the impact failure process. However, a ductile–brittle transition process occurred with less plastic deformation of the matrix for the notched impact-fractured surfaces of the PLA/EUG/OMT (85/15/4) and (85/15/6) samples (see Figure 10E,F). This indicates a rapid decline in the toughness when the OMMT content is equal to or greater than 4 phr. This is highly consistent with the relationship seen between the notched impact strength and the OMMT content. Possible reasons for this could be as follows: When the OMMT content was ≥4 phr, the average size of the EUG particles reduced to far less than 1 μm. This resulted in an excessive stress concentration in the PLA matrix. Moreover, with the excessive addition of OMMT, the aggregation of the OMMT nanoparticles in the PLA/EUG/OMMT blending system became inevitable. The OMMT’s aggregation and the excessive stress concentration resulted in the rapid formation and growth of micro-cracks in the matrix, accompanied by less plastic deformation. Similar conclusions were also reported in previous references to PLA/PU/SiO_2_ blends [42,43], wherein the selective localization of SiO_2_ nanoparticles improved the interfacial compatibilization and tailored the micro-morphology of the PLA/PU blend. Thus, a series of PLA/PU blends with higher toughness was prepared by adding appropriate amounts of SiO_2_ nanoparticles. However, the authors pointed out that on further increasing the SiO_2_ content, aggregates of SiO_2_ nanoparticles formed in the matrix, which accelerated crack initiation and propagation and weakened the toughness.

From the aforementioned test results on the mechanical performance, it is evident that the PLA/EUG (85/15) blend containing 2 phr of OMMT exhibits a significant enhancement in its tensile toughness and impact toughness. Simultaneously, its tensile yield strength and tensile modulus can achieve values of 32.4 MPa and 1451.1 MPa, respectively. Consequently, its mechanical properties are comparable to those of polypropylene materials [44,45].

### 3.3. Crystalline Properties

It is well known that the crystal properties of a polymer blend are important attributes which significantly influence its mechanical performance. In this study, DSC and XRD were used to determine the crystallinity and the crystal structure of the PLA/EUG/OMMT samples with varying OMMT contents.

**Figure 11:**
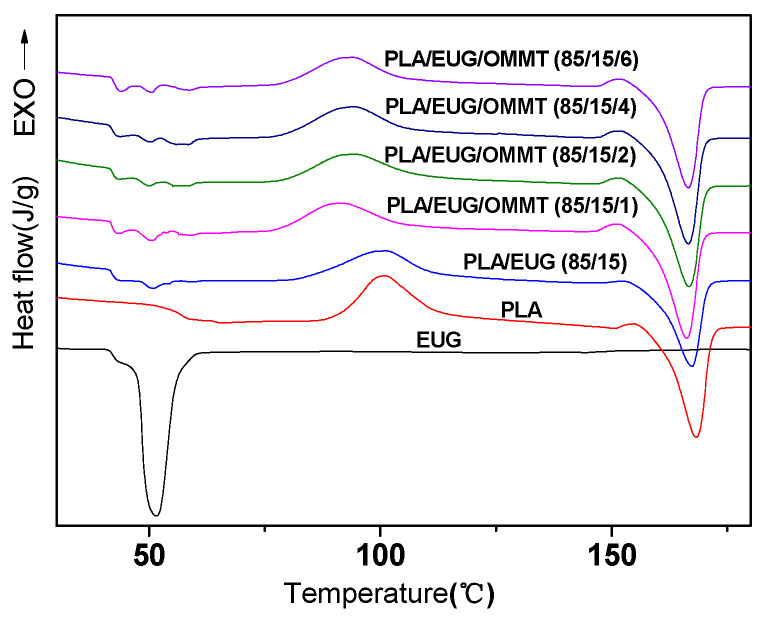
Heating DSC thermograms of neat PLA, EUG, and PLA/EUG/OMMT (85/15/x) samples.

Figure 11 shows the heating DSC thermograms of the PLA/EUG/OMMT (85/15/x) samples with varying OMMT contents. For comparison, DSC thermograms of neat PLA and neat EGU are also shown in Figure 11. The thermodynamic parameters of the PLA matrix and the EUG component are listed in Table 5 and Table 6, respectively.

As seen in Figure 11, the neat PLA sample, on heating from 20 °C to 200 °C, underwent the following phase transformations: a cold crystallization process [46] ($T_{cc1}$ = 101.0 °C); an exothermic process during the transition from an imperfect *α*′ crystal form to a more perfect *α* crystal form [47] ($T_{cc1}^{\prime }$ = 155.0 °C); and a melting process ($T_{m1}$ = 168.3 °C). In contrast, the neat EUG sample underwent a melting endothermic process with the appearance of two melting peaks at 43.2 °C and 51.7 °C. Similar to the neat PLA and neat EUG samples, the PLA/EUG and PLA/EUG/OMMT samples also exhibited similar phase transitions, as shown as Figure 11.

The crystallinity ($X_{c1}$) of the PLA matrix can be calculated from Equation (8).

(8) $$X_{c1}=\frac{\left|\Delta H_{m1}\right|\;-\left|\Delta H_{\mathrm{cc}1}\right|-\left|{\Delta H_{cc1}}^{\prime }\right|}{\omega_{PLA}\times \left|\Delta H_{m1}^{0}\right|}$$

where $\Delta H_{m1}$ and $\Delta H_{\mathrm{cc}1}\;$ are, respectively, the melting enthalpy and the cold crystallization enthalpy of the PLA matrix, ${\Delta H_{cc1}}^{\prime }$ is the exothermic enthalpy during the transformation from the imperfect *α*′ crystal form to a more perfect *α* crystal form, $\omega_{PLA}$ is the mass ratio of PLA in the samples, and $\Delta H_{m1}^{0}$ is the ideal melting enthalpy of 100% crystalline PLA (−93.6 J/g) [48]. In this paper, the endothermic enthalpy has a negative value in the DSC measurements.

The crystallinity ($X_{c2}$) of the EUG component can be calculated using Equation (9).

(9) $$X_{c2}=\frac{\left|\Delta H_{m2}\right|}{\omega_{EUG}\times \left|\Delta H_{m2}^{0}\right|}$$

where $\Delta H_{m2}$ is the melting enthalpy of EUG, $\omega_{EUG}$ is the mass ratio of EUG in the samples, and $\Delta H_{m2}^{0}$ is the ideal melting enthalpy of 100% crystalline EUG (−186.8 J/g) [49].

The crystallinity of the neat PLA sample is only 3.0% in Table 5, indicating that PLA is almost amorphous. This can be attributed to the slow melt-crystallization rate of PLA. Similar to neat PLA, the PLA matrix of the binary PLA/EUG sample also exhibits very low crystallinity (ca. 4.1%). This indicates that the addition of EUG cannot promote the crystallization of the PLA matrix.

It is noteworthy that the crystallinity of the PLA matrix exhibits a slight increase as the amount of OMMT increases. For instance, the crystallinity of the PLA matrix in the PLA/EUG/OMMT (85/15/6) sample increases from 4.1% for the PLA/EUG (85/15) sample to 10.4%. This can be attributed to the heterogeneous nucleation effect of the OMMT nanoparticles in the PLA matrix to some extent [50]. Overall, it can be concluded that the introduction of EUG and OMMT components has much less impact on the crystallinity of the PLA matrix.

Equally, it is evident from Table 6 that the crystallinities of the EUG components in the PLA/EUG (85/15) and PLA/EUG/OMMT (85/15/x) samples are very small (*X*_c_ ≤ 9.6%), far smaller than the crystallinity of the neat EUG sample (*X*_c_ = 24.4%). It is proposed that the crystallization ability of the EUG-rich domains may be disturbed and restricted by the PLA chains.

**Figure 12:**
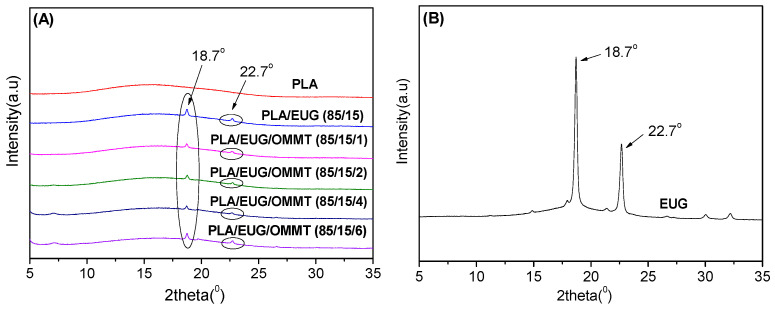
XRD patterns of PLA/EUG/OMMT (85/15/x) samples (**A**) and neat EUG samples (**B**).

XRD was used to further investigate the crystalline structures of the PLA/EUG/OMMT (85/15/x) samples, and their XRD patterns are shown in Figure 12. In Figure 12, the XRD pattern of the neat PLA sample shows a weak and broader peak, indicating its amorphous nature [51]. In contrast, the XRD pattern of the neat EUG sample shows two strong diffraction peaks at 2*θ* = 18.7° and 22.7°, corresponding to the (120) and (200) planes of the *β* crystalline form, respectively [52]. This implies that neat EUG is a semi-crystalline polymer with higher crystallinity. The XRD results are consistent with the findings of the DSC analysis presented in Table 5 and Table 6. The XRD patterns of the PLA/EUG samples with and without OMMT show only two diffraction peaks with significantly weakened intensities at 2*θ* = 18.7° and 22.7°. They can be attributed to the diffractions of the semi-crystalline EUG component. As discussed in the DSC analysis, the decrease in the crystallinity, coupled with the weakening of the diffraction peaks of the EUG component, can be attributed to the fact that the crystallization ability of the EUG-rich domains is disturbed and restricted by the PLA chains. Further, it should be noted that the characteristic diffraction peaks of PLA crystals are absent in the XRD patterns of the PLA/EUG samples with and without OMMT. However, the DSC analysis reveals that increasing the OMMT content from 0 phr to 6 phr results in a rise in the crystallinity of the PLA matrix, from 4.1% to 10.4%. The absence of the diffraction peaks for the PLA crystals can be attributed to the fact that the crystalline regions in the PLA matrix of the blend samples are very imperfect, however, and no obvious X-ray diffraction can be generated and detected effectively.

It is concluded from the DSC and XRD results that the addition of EUG and OMMT has a minimal impact on the crystallinity and crystalline structure of the PLA matrix. The crystallinities of the PLA matrix in the PLA/EUG (85/15) samples with and without OMMT do not exceed 10.4%, indicating that such low crystallinity levels are unlikely to significantly influence the mechanical properties of the blend samples.

### 3.4. Thermal Degradation Behavior

**Figure 13:**
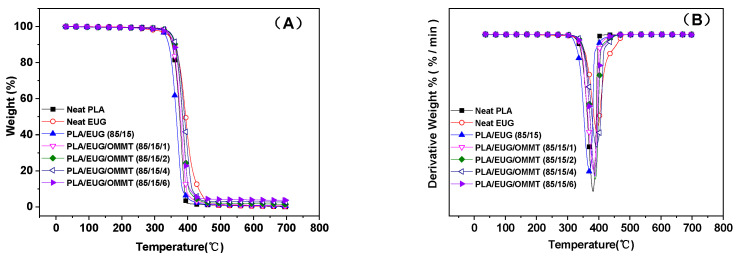
TGA curves (**A**) and DTG curves (**B**) for neat PLA, EUG, and PLA/EUG/OMMT (85/15/x) samples with various OMMT contents.

A TGA was conducted to investigate the thermal degradation behaviors of the PLA/EUG and PLA/EUG/OMMT blends with varying OMMT contents. Figure 13A,B show the TGA curves of the samples and their corresponding differential thermogravimetric (DTG) curves, respectively. From the TGA curves, the temperatures corresponding to 5% and 50% weight losses from the samples were determined and noted as *T*_−5 wt%_ and *T*_−50 wt%_, respectively. *T*_−5 wt%_ is considered to be the initial thermal degradation temperature. The temperatures corresponding to the maximum decomposition rate *T*_max_ were obtained from the peak values in the DTG curves and are listed in Table 7. The *T*_−5 wt%_, *T*_−50 wt%_, and *T*_max_ values of the neat PLA sample were 343.8 °C, 375.7 °C, and 381.7 °C, respectively. The thermal degradation of PLA was very complex, involving various non-radical and free radical reactions, such as random chain scission reactions, depolymerization, oxidative degradation, intra- and inter-molecular transesterification reactions, hydrolysis, pyrolysis, elimination, free radical reactions, etc. However, the most commonly occurring reaction was intramolecular transesterification to form cyclic oligomers and lactide or lactic acid [53,54]. It is evident from Table 7 that EUG had thermal decomposition temperatures closer to those of PLA, wherein the *T*_−5 wt%_, *T*_−50 wt%_, and *T*_max_ values were 342.7 °C, 394.0 °C, and 394.6 °C, respectively. However, there are no reports in the literature illustrating the thermal degradation process for EUG resin. Chen et al. [55] studied the thermal cleavage of NR and found that the cleavage reaction of NR was mainly the result of C-C single bond cleavage adjacent to the double bond on the main chain. This resulted in the depolymerization and then the formation of free radicals, with the generation of dipentene and isoprene as the main products. EUG refers to *trans*-1,4-polyisoprene, which is an isomer of NR, and so it can be inferred that it may undergo a similar thermal cracking process. Obviously, the EUG carbon chains and PLA polyester chains experience different thermal degradation mechanisms. Thus, the thermal degradation mechanisms become more complicated for blends of the two.

In Figure 13, the TGA curve for the PLA/EUG (85/15) sample shows only a single step of weight loss that can be attributed to the overlapping of the weight loss steps for the PLA matrix and the EUG component. It is seen from Table 7 that compared with neat PLA and neat EUG, the thermal stability of the PLA/EUG (85/15) sample decreases to some extent, wherein its *T*_−5 wt%_, *T*_−50 wt%_, and *T*_max_ values are 332.9 °C, 365.5 °C, and 369.8 °C, respectively. However, with an increase in the OMMT content, the PLA/EUG/OMMT samples show improved thermal stability. For instance, on introducing 2 phr of OMMT into the PLA/EUG blend, the *T*_−5 wt%_ value rises from 332.9 °C for the PLA/EUG (85/15) sample to 351.3 °C. When the OMMT content further increases to 4 phr, the *T*_−5 wt%_ value rises by approximately 20 °C relative to that of the PLA/EUG (85/15) sample. This change can be attributed to the barrier effect of OMMT, wherein the lamellar structure of OMMT plays a key role in obstructing the diffusion of the volatile decomposition products formed during decomposition at elevated temperatures. This results in enhanced thermal stability of the PLA/EUG/OMMT samples. This finding aligns with the thermal stabilities reported for PLA/halloysite and OMMT/graphene–PLA/PCL nano-blend materials [56,57]. On the other hand, during thermal degradation, OMMT can form acidic sites on its surface, which facilitate cross-linking reactions between polymer chains. This process will result in the formation of a physically and chemically cross-linked network structure, consequently enhancing the thermal stability of the blends. Qin et al. [58] previously reported such a phenomenon in a PP/clay blend.

### 3.5. Extrudate Swell Behavior

Extrusion is an important method for processing and molding in polymer processing engineering, and it is useful for the preparation of various types of polymer-based products, such as pipes, sheets, bars, profiles, etc. However, the recoverable elastic deformation of the viscoelastic polymer melt during its flow can result in an extrusion swelling phenomenon. Herein, the cross-sectional sizes and shapes of the extrudates are inconsistent with the cross-sectional sizes and shapes of the extrusion dies. Normally, extrudate swelling causes serious harm to the dimensional accuracy, quality, and appearance of the products obtained from the polymer extrudate. Hence, it is necessary to find ways to restrain or reduce extrudate swelling phenomena in polymer materials.

**Figure 14:**
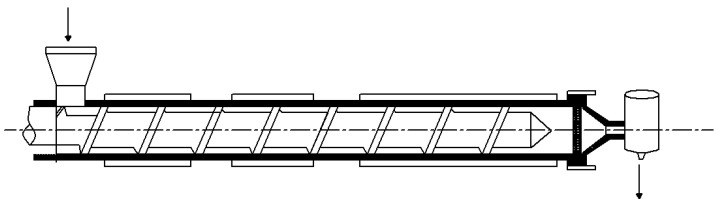
Schematic diagram of the extrusion swelling experiment device.

**Figure 15:**
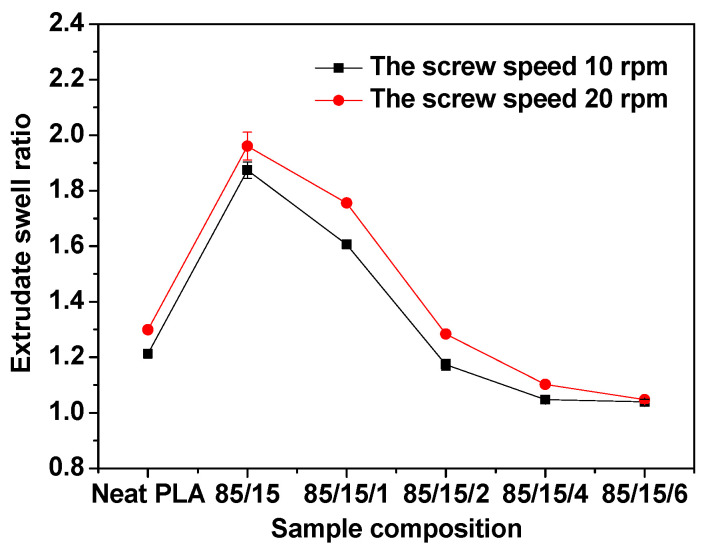
Extrudate swell ratios for neat PLA and PLA/EUG/OMMT (85/15/x) samples.

**Figure 16:**
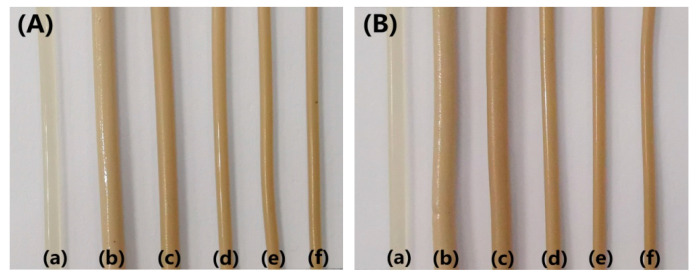
Pictures of extrudates obtained at different screw speeds of (**A**) 10 rpm and (**B**) 20 rpm for (**a**) neat PLA, (**b**) PLA/EUG (85/15), (**c**) PLA/EUG/OMMT (85/15/1), (**d**) PLA/EUG/OMMT (85/15/2), (**e**) PLA/EUG/OMMT (85/15/4), and (**f**) PLA/EUG/OMMT (85/15/6).

In this study, the extrudate swell behaviors of neat PLA and the PLA/EUG blends with and without OMMT were evaluated using a single extruder, equipped with a capillary die (capillary diameter = 1.27 mm; L/D ratio = 20:1). Figure 14 shows a schematic diagram of the experimental set-up for the extrudate swell testing. Figure 15 shows the extrudate swell ratios of the samples at a processing temperature of 190 °C, wherein the screw speeds were maintained at 10 rpm and 20 rpm. Pictures of the extrudates are shown in Figure 16. It can be seen in Figure 15 that the extrudate swell ratio of the binary PLA/EUG sample is significantly higher than that of the neat PLA sample. The extrudate swell ratio of the binary sample increases from 1.21 for the neat PLA sample to 1.87 at a screw speed of 10 rpm, representing an approximate increase of 55%. A significant increase in the extrudate swell ratio will markedly diminish the dimensional stability of the PLA/EUG extrudate. This is mainly attributed to the incorporation of a highly elastic EUG component, which increases the viscoelasticity of the binary PLA/EUG melt. Thus, it greatly prolongs the elastic recovery time of the PLA/EUG melt (significantly longer than the residence time of the melt in the capillary tubes), which results in a substantial increase in the extrudate swell ratio of the melt. However, the extrudate swell ratio of the blend decreases significantly upon the addition of OMMT. For instance, when the OMMT content increases to 2 phr, the extrudate swell ratio decreases to 1.17, slightly lower than that of neat PLA (1.21). Upon further increasing the OMMT content to 4 phr, the extrudate swell ratio decreases further to 1.05. Obviously, the incorporation of OMMT markedly mitigates the extrudate swell behavior in the PLA/EUG blend, thereby enhancing the dimensional stability of the products obtained through extrusion molding. Taking the TEM images of the PLA/EUG/OMMT (85/15x) blend, it is believed that the large number of selectively distributed OMMT nanolayers at the PLA/EUG interface can effectively constrain the mobility of the EUG macromolecular chains and segments. This has a significant inhibitory effect on the recovery of the elastic deformation of the EUG domains, where the elastic deformation is a consequence of the extrusion flow process in the capillary die [59]. Moreover, the dispersion of a small amount of OMMT in the PLA matrix is unavoidable, which contributes to reducing the extrudate swell behavior of the PLA matrix to a certain extent. In this circumstance, the extrudate swell behavior of the PLA/EUG blend can be significantly weakened upon the addition of OMMT. Furthermore, it is confirmed from Figure 15 that the extrudate swell ratio increases with an increase in the screw speed from 10 rpm to 20 rpm. This phenomenon can be attributed to the reduced residence time of the melt in the capillary die channel as the flow rate increases at a given temperature during the extrusion process. Stress relaxation will not occur if the residence time is shorter than the relaxation time of the molecular chains in the melt. The elastic recovery of the deformation produced in the flow increases correspondingly in this situation, which leads to an increase in the extrudate swell of the melt [60,61]. The results of the extrudate swell experiment prove that the introduction of OMMT can effectively reduce the extrudate swell behavior of the PLA/EUG/OMMT blends.

## 4. Conclusions

In summary, a highly toughened bio-based PLA/EUG blend was successfully prepared using OMMT as the compatibilizer. The theoretically calculated interfacial energy shows that the OMMT nanolayers are selectively positioned at the PLA/EUG interface. This selective localization of OMMT is proven further through a TEM analysis. The SEM images show that the EUG domain size decreases significantly with an increase in the OMMT content. This is associated with a reduction in the interfacial tension and the inhibition of coalescence of the EUG domains by the OMMT layers distributed at the interface. As the OMMT content increased from 0 phr to 2 phr, the notched Izod impact strength of the PLA/EUG/OMMT (85/15/2) blend reaches a maximum value of 44.6 kJ/m^2^, significantly higher than the values of 3.8 kJ/m^2^ for neat PLA and 4.7 kJ/m^2^ for the PLA/EUG (85/15) blend. In addition, the PLA/EUG/OMMT blend demonstrates a substantial improvement in tensile ductility when an appropriate amount of OMMT is added. The elongation at break of the PLA/EUG/OMMT (85/15/2) blend reaches a maximum value of 102.8%, significantly higher than the values of 5.3% for neat PLA and 18.8% for the PLA/EUG (85/15) blend. It is believed that the enhanced toughness of the PLA/EUG/OMMT blends can primarily be attributed to the refinement and more uniform dispersion of the EUG domains, which is caused by the incorporation of OMMT. Simultaneously, the tensile yield strength and the tensile modulus of the PLA/EUG/OMMT (85/15/2) blend can achieve values of 32.4 MPa and 1451.1 MPa, respectively. Consequently, its mechanical properties are comparable to those of polypropylene materials. The results of the DSC and XRD analyses indicate that the incorporation of EUG and OMMT has a minimal impact on the crystallinity or the crystalline structure of the PLA matrix. The crystallinity of the PLA matrixes in all of the PLA/EUG blends with and without OMMT does not exceed 10.4%. With an increase in the OMMT content, the thermal stability of the PLA/EUG/OMMT blends obviously improves due to the barrier effect of the OMMT nanolayers. With the incorporation of 2 phr of OMMT into the PLA/EUG blend, the *T*_−5 wt%_ value increases from 332.9 °C for the PLA/EUG (85/15) blend to 351.3 °C. When the OMMT content further increases to 4 phr, the *T*_−5 wt%_ value rises by approximately 20 °C compared to its value in the PLA/EUG (85/15) blend. Notably, the incorporation of OMMT significantly reduces the extrudate swell behavior of the PLA/EUG blends. When the OMMT content increases to 2 phr, the extrudate swell ratio decreases to 1.17, which is significantly lower than that of the neat PLA/EUG (85/15) blend (1.87) and slightly lower than that of neat PLA (1.21), which is conducive for controlling the dimensional stability of the products obtained through extrusion molding.

In this study, the PLA/EUG (85/15) blend containing 2 phr of OMMT exhibited more balanced performance characteristics; in particular, its mechanical properties were comparable to those of PP materials. Therefore, it will have promising application prospects in the field of eco-friendly and renewable general plastic products, such as shopping bags, packaging films, tableware, plastic parts for toys, electrical appliance shells, etc.

## Figures and Tables

**Table 1:** Mass ratios of PLA/EUG/OMMT blends.

Sample	PLA (phr)	EUG (phr)	OMMT (phr)
PLA/EUG (85/15)	85	15	0
PLA/EUG/OMMT (85/15/1)	85	15	1
PLA/EUG/OMMT (85/15/2)	85	15	2
PLA/EUG/OMMT (85/15/4)	85	15	4
PLA/EUG/OMMT (85/15/6)	85	15	6

**Table 2:** Measured contact angles and calculated surface energies of various samples.

Samples	Contact Angle (°)		Surface Energy at 25 °C (mJ/m^2^)			Temp. Coefficient –d*γ*/d*T*	Surface Energy at 190 °C (mJ/m^2^)		
	H_2_O	CH_2_I_2_	$\gamma_{s}^{d}$	$\gamma_{s}^{p}$	γ_s_		$\gamma_{s}^{d}$	$\gamma_{s}^{p}$	γ_s_
PLA	67.5 ± 2.5	42.6 ± 2.1	31.4	11.2	42.6	0.05 [39]	25.3	9.1	34.4
EUG	83.5 ± 2.3	65.7 ± 2.3	20.9	7.1	28.0	0.05 [39]	14.7	5.0	19.7
OMMT			31.5	11.1	42.5 [40]	0.1 [39]	19.2	6.8	26.0
H_2_O			22.5	50.3	72.8 [41]				
CH_2_I_2_			48.5	2.3	50.8 [41]				

**Table 3:** Calculated interfacial energy and wetting coefficients according to harmonic-mean and geometric-mean equations.

Samples	Interfacial Energy (mJ/m^2^)		Wetting Coefficient, ω_a_	
	Harmonic-Mean Equation	Geometric-Mean Equation	Harmonic-Mean Equation	Geometric-Mean Equation
PLA/OMMT	1.17	0.59		
EUG/OMMT	0.87	0.44	0.07	0.07
PLA/EUG	3.99	2.03		

**Table 4:** Tensile properties of PLA/EUG/OMMT (85/15/x) samples.

Samples	Tensile Yield Strength (MPa)	Tensile Modulus (MPa)	Elongation at Break (%)
Neat PLA	56.2 ± 3.8	1698.5 ± 85.1	5.3 ± 0.7
PLA/EUG (85/15)	36.1 ± 0.9	1432.2 ± 75.6	18.8 ± 3.3
PLA/EUG/OMMT(85/15/1)	33.9 ± 0.7	1475.9 ± 51.3	96.6 ± 26.6
PLA/EUG/OMMT(85/15/2)	32.4 ± 0.9	1451.1 ± 72.7	102.8 ± 32.0
PLA/EUG/OMMT(85/15/4)	31.8 ± 0.7	1484.0 ± 40.0	37.3 ± 9.2
PLA/EUG/OMMT(85/15/6)	28.6 ± 1.3	1437.6 ± 36.6	19.8 ± 8.0

**Table 5:** DSC parameters of PLA matrix of PLA/EUG/OMMT (85/15/x) samples.

Samples	$T_{cc1}$	$\Delta H_{cc1}$	$T_{cc1}^{\prime }$	${\Delta H_{cc1}}^{\prime }$	$T_{m1}$	$\Delta H_{m1}$	$X_{c1}$
	(°C)	(J·g^−1^)	(°C)	(J·g^−1^)	(°C)	(J·g^−1^)	(%)
Neat PLA	101.0	28.02	155.0	0.46	168.3	−31.3	3.0
PLA/EUG (85/15)	100.9	21.85	152.2	0.21	167.5	−25.35	4.1
PLA/EUG/OMMT(85/15/1)	91.5	20.31	151.5	1.71	166.2	−29.61	9.6
PLA/EUG/OMMT(85/15/2)	94.2	20.64	151.9	1.31	166.7	−29.43	9.6
PLA/EUG/OMMT(85/15/4)	94.0	21.07	151.8	1.33	166.5	−29.54	9.3
PLA/EUG/OMMT(85/15/6)	93.8	19.09	151.6	1.27	166.5	−28.16	10.4

**Table 6:** DSC parameters of EUG component of PLA/EUG/OMMT (85/15/x) samples.

Samples	$T_{m2}^{1}$ (°C)	$T_{m2}^{2}$ (°C)	$\Delta H_{m2}$ (J·g^−1^)	$X_{c2}$ (%)
Neat EUG	43.2	51.7	−45.55	24.4
PLA/EUG (85/15)	43.1	50.7	−2.70	9.6
PLA/EUG/OMMT (85/15/1)	43.0	50.1	−2.43	8.8
PLA/EUG/OMMT (85/15/2)	43.0	50.0	−2.25	8.2
PLA/EUG/OMMT (85/15/4)	43.0	50.0	−2.09	7.8
PLA/EUG/OMMT (85/15/6)	43.5	50.3	−1.95	7.4

**Table 7:** TGA data for neat PLA, EUG, and PLA/EUG/OMMT (85/15/x) blends.

Samples	*T*_−5 wt%_	*T*_−50 wt%_	*T*_max_
	(°C)	(°C)	(°C)
Neat PLA	343.8	375.7	381.7
Neat EUG	342.7	394.0	394.6
PLA/EUG (85/15)	332.9	365.5	369.8
PLA/EUG/OMMT (85/15/1)	346.8	378.5	382.4
PLA/EUG/OMMT(85/15/2)	351.3	385.0	388.6
PLA/EUG/OMMT(85/15/4)	353.1	390.4	391.7
PLA/EUG/OMMT(85/15/6)	346.4	384.0	387.3

## Data Availability

The data presented in this study are available on request from the corresponding author and the first author.
